# Clinicopathologic data of individuals with oral lichen planus: A Brazilian case series

**DOI:** 10.4317/jced.56379

**Published:** 2019-12-01

**Authors:** Sara-Lia-Gonçalves de Lima, José-Alcides-Almeida de Arruda, Lucas-Guimarães Abreu, Ricardo-Alves Mesquita, Rejane-Faria Ribeiro-Rotta, Elismauro-Francisco Mendonça, Diego-Antônio-Costa Arantes, Aline-Carvalho Batista

**Affiliations:** 1 DDS, MSc Student, Department of Stomatology (Oral Medicine and Oral Pathology), School of Dentistry, Universidade Federal de Goiás, Goiânia, GO, Brazil; 2 DDS, MSc, PhD Student, Department of Oral Surgery and Pathology, School of Dentistry, Universidade Federal de Minas Gerais, Belo Horizonte, MG, Brazil; 3 DDS, PhD, Department of Pediatric Dentistry and Orthodontics, School of Dentistry, Universidade Federal de Minas Gerais, Belo Horizonte, MG, Brazil; 4 DDS, PhD, Department of Oral Surgery and Pathology, School of Dentistry, Universidade Federal de Minas Gerais, Belo Horizonte, MG, Brazil; 5 DDS, PhD, Department of Stomatology (Oral Medicine and Oral Pathology), School of Dentistry, Universidade Federal de Goiás, Goiânia, GO, Brazil

## Abstract

**Background:**

The aim of the present series was to analyze the sociodemographic characteristics, clinicopathologic features, and oral health-related quality of life of 41 individuals with oral lichen planus (OLP).

**Material and Methods:**

In a retrospective analysis (1998-2018), individuals with a clinical diagnosis of OLP from a referral service of Oral Medicine of Brazil were invited for follow-up. The individuals were assessed using the Oral Health Impact Profile-14 (OHIP-14) form. Histopathological data were reviewed according to the latest criteria proposed by the American Academy of Oral and Maxillofacial Pathology (AAOMP/2016).

**Results:**

This series mainly consisted of females (70.7%) in their forties (31.7%). The buccal mucosa (68.2%) was the most commonly affected site. Reticular (56.1%) and erosive (34.3%) appearances were the most frequent. According to OHIP-14, individuals with OLP at multiple sites in the oral cavity showed worse values in the handicap domain and those who did not respond to corticosteroids showed a higher score on the psychological discomfort domain.

**Conclusions:**

The findings of the present study, using the AAOMP/2016 criteria, agree with case series and retrospective studies reported in the literature. Besides, OLP in its more severe clinical forms had an influence on patient quality of life.

** Key words:**Diagnosis, epidemiology, oral lichen planus, oral mucosa, quality of life.

## Introduction

The first clinical characterization of oral lichen planus (OLP) was described by Dr. Erasmus Wilson in 1866 (1) as a nonscrapable white plaque found in the buccal mucosa, especially in middle-aged women. Later, in 1895, Louis Wickham added to this description a pattern of interlaced white striae, which were then called Wickham striae (2). Over a period of 40 years, OLP was studied and diagnosed only in terms of its clinical characteristics, since microscopic findings were only defined for diagnosis in 1906 by M. William Dubreuilh (3).

Approximately 15% of individuals with OLP may also exhibit skin lesions (4). OLP individuals may be asymptomatic or may suffer extreme degrees of pain with a possible impact on their quality of life (5,6). OLP generally affects the buccal mucosa, gingiva, and tongue, manifesting in the form of bilateral and symmetric lesions with a reticular pattern, with the erosive, atrophic, bullous and plaque-like types being accepted only in the presence of reticular lesions (7). The American Academy of Oral and Maxillofacial Pathology (AAOMP/2016) (5) proposed a modification in the clinical and morphological criteria described by the World Health Organization in 1978 (7), and reviewed by van der Meij and van der Waal in 2003 (8), characterizing OLP as white or red lesion, multifocal, of symmetric distribution, being classified as reticular/papular, atrophic (erythematous), erosive (ulcerative), plaque and bullous (5). In addition, classical histopathological characteristics such as the presence of an infiltrate predominantly consisting of lymphocytes distributed as a band in the subepithelial region, lymphocyte exocytosis and basal keratinocyte liquefaction have been well established and, according to the AAOMP/2016, should be associated with the clinical characteristics for a final diagnosis (5).

This inflammatory disease of an autoimmune nature regulated by T lymphocytes affects approximately 2% of the world population, mainly occurring in women aged 30 to 80 years with mean of 52 years (3,5). Its etiopathogeny is still unknown and patient treatment has been limited only to symptom relief with topical or systemic corticosteroids (9,10). A relationship between autoimmune diseases and stress, anxiety and depression is described in some studies (11,12) and, among these diseases, a possible relationship has been reported between emotional changes and the manifestation of OLP with an impact on the quality of life of individuals (6,11,13,14).

The Oral Health Impact Profile-14 (OHIP-14) is used to measure the quality of life of individuals with OLP or with other autoimmune diseases affecting the oral region (13-15). The results obtained have pointed out the importance of this instrument for the understanding of the impact of autoimmune diseases on quality of life; however, few studies using OHIP-14 for these diseases have been conducted on the Brazilian population (15).

Although the clinical and morphological characteristics of OLP are well defined, there is a challenge in its histopathological diagnosis due to the lack of clinical data submitted to oral and maxillofacial pathology services, with an increased risk of confusion with other lesions, such as oral leukoplakia, frictional keratosis and lichenoid reaction (5,16). Case series studies of Asian, American, European and Oceanic populations have reported epidemiological data of individuals with OLP (17-20). However, few investigations have described the occurrence and clinicopathologic features of these lesions in Brazil (21-23). Therefore, the purpose of the present study was to report 41 cases of OLP according to the clinicopathologic criteria proposed by AAOMP/2016. In addition, the patients were invited to engage in follow-up and were evaluated by OHIP-14 in order to correlate the clinical features of the disease with the quality of life of these affected individuals.

## Material and Methods

-Case series and ethical issues

A retrospective analysis was conducted on 41 individuals with OLP. The guidelines proposed to strengthen the description of observational studies (STROBE) were followed (24). The present study consisted of individuals who had been referred to the service of Oral and Maxillofacial Pathology and Oral Medicine (CGDB) of School of Dentistry of Universidade Federal de Goiás (UFG), Goiânia, Brazil between 1998 and 2018. During this 20-year period, the participants had been evaluated and clinical information had been compiled by various providers with experience in oral diagnosis. The study was approved by the Ethics Committee on Human Research of the institution (No. 3.095.226). According to the Statement of Helsinki, the participants agreed with the publication of their cases.

-Data collection

**Figure 1:**
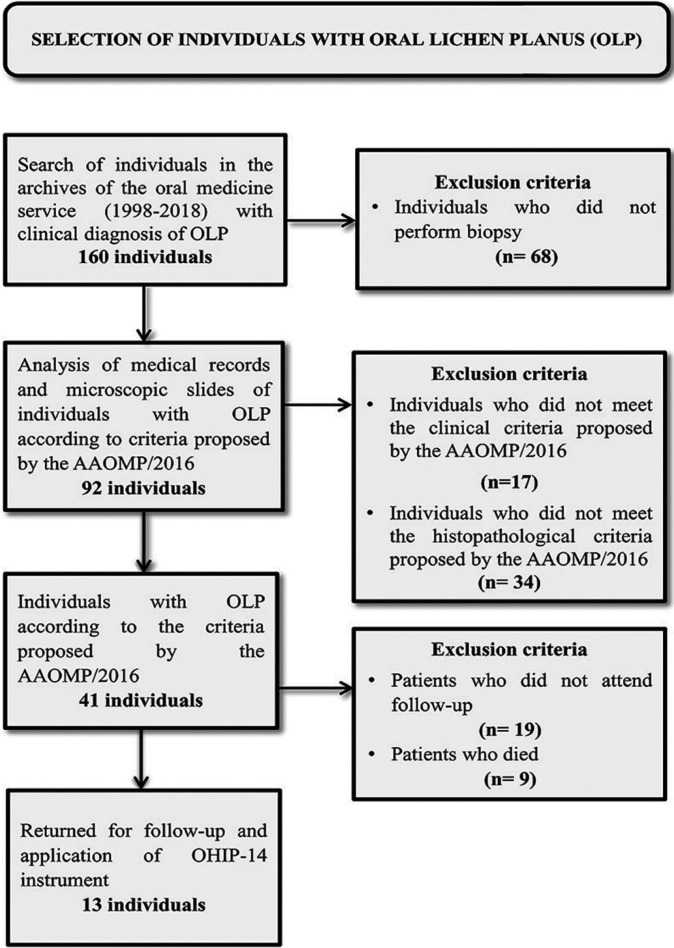
Sample selection flowchart.

The case series is reported according to the flow diagram presented in Figure 1. The demographic data (age and sex) of the selected subjects were obtained by an active survey and evaluation of the medical records in the archives of the service. The clinical data recorded were also evaluated in terms of the aspect of the lesions: ulceration, whitish striae, and white plaques. The lesions were then classified as reticular, erosive, atrophic or bullous. Also, were divided into groups according to their distribution as follows: lesion in only single bilateral/symmetric site and multiple bilateral/symmetric sites. The following anatomical locations were possible: buccal mucosa, tongue, lips, gingiva, and palate. Regarding patient treatment, two therapeutic modalities were established: mouthwash/oral use of dexamethasone, 0.1 mg/mL, 12/12 hours and topical application of triamcinolone acetonide, 1 mg/g, 8/8 hours. Data about lesion recurrence were collected and the following groups were defined according to time of recurrence: lesions recurring after 0-1 month, 2-3 months, 4-6 months, one year, and more than one year ( Table 1).

**Table 1:**
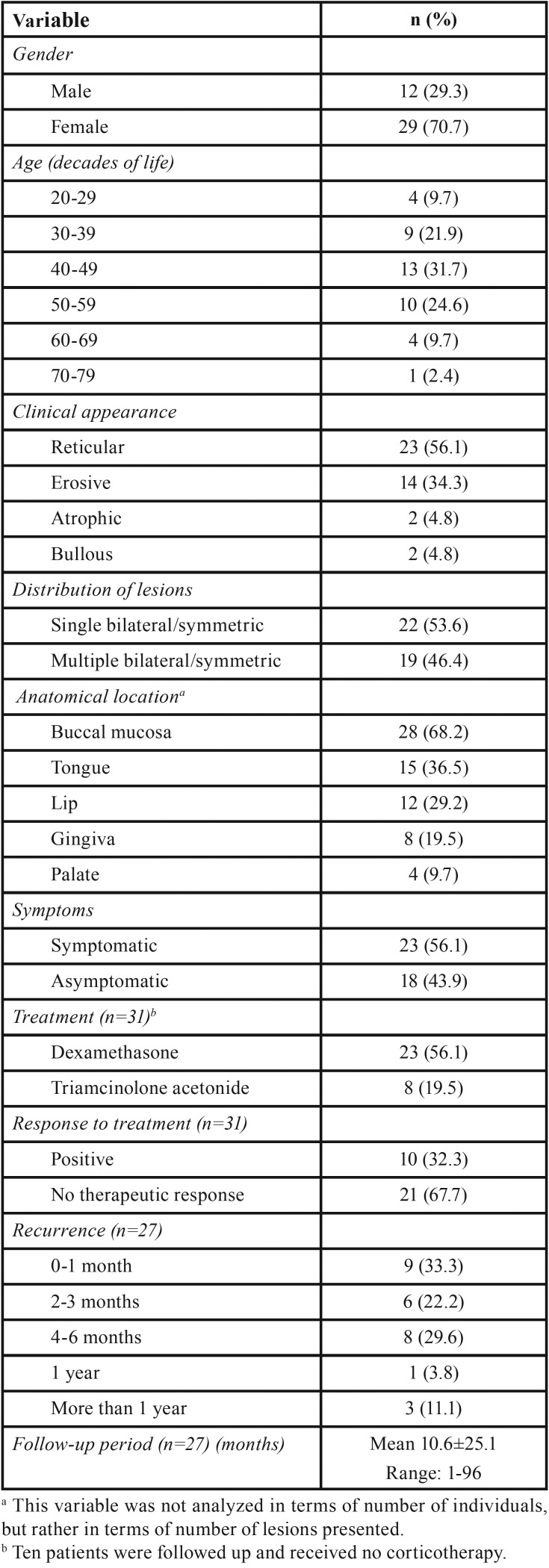
Demographic data and clinical features of the cases of oral lichen planus.

In order to confirm the diagnosis of OLP, the records of the clinical and histopathological parameters of the individuals were reviewed according to the criteria proposed by AAOMP/2016 (5). Clinically, lesions with a confirmed diagnosis of OLP had to be obligatorily symmetric and multifocal, consisting of the exclusively reticular form or of the atrophic, erosive, bullous and/or plaque forms. The histopathological records of all participants from the Oral Pathology service of the UFG were also reviewed in order to microscopically confirm the cases of OLP. Four consultants (A.C.B., E.F.M., D.A.C.A., and R.A.M.) in Oral and Maxillofacial Pathology and Oral Medicine assisted with the ratification of the cases.

The exclusion criteria were: missing information regarding sociodemographic data and the clinicopathologic characteristics of the disease, the presence of unilateral lesions without an ulcerated, whitish, atrophic or bullous aspect, lesions in contact with amalgam or prosthetic materials or a history of tobacco, alcohol or cinnamon use.

The histopathological data were supposed to show a predominantly lymphocytic infiltrate arranged in a band and located on the lamina propria, with destruction/liquefaction of basal keratinocytes, lymphocyte exocytosis, and absence of epithelial dysplasia (5). Exclusion criteria are shown in Figure 1.

In addition, to confirmatory and illustrative purposes, the immunohistochemical analyses for recognition of CD8+ T lymphocytes (clone C8/144B; Dako, Carpinteria, CA, USA; 1:200) was accomplished in all samples. The sections were treated with the Kit Novolink™ Max Polymer Detection System (Novocastra, Leica Biosystems Gmb, Wetzlar, HE, Germany) and the reactions were developed with 3.3′-diaminobenzidine (DAB, Dako, Carpinteria, CA, USA).

-OHIP-14 questionnaire

The OHIP-14 questionnaire, first developed in English, was adapted and validated for Brazilian Portuguese (25) and was used for the assessment of the impact of oral conditions on patient quality of life. The questionnaire contains 14 questions divided into seven domains: functional limitation, physical pain, psychological discomfort, physical disability, psychological disability, social disability, and handicap. Five response options are available for each question, with the following scores: ‘never’ = 0, ‘hardly ever’ = 1, ‘sometimes’ = 2, ‘fairly often’ = 3, ‘very often’ = 4. The total score ranges from 0 to 56 points. The higher the score, the greater the negative perception of the patient regarding the impact of his oral conditions on his quality of life. Scores for the seven domains were also possible (25). The patients were invited by telephone to engage in follow-up and were evaluated by OHIP-14.

-Statistical analysis

Data were analyzed statistically using the Statistical Package for the Social Sciences (SPSS, SPSS Inc., version 23.0, Armonk, USA). A descriptive analysis was carried out using the absolute and relative values of the clinicodemographic data collected. The sample was divided into groups according to the characteristics of the lesions, i.e., clinical type (reticular and erosive), location (a single bilateral site and multiple bilateral sites), and according to the response to topical or systemic corticotherapy (positive or no response). The Shapiro-Wilk test was used to determine the normality of the OHIP-14 scores and the Mann-Whitney test was used to compare the groups regarding the OHIP-14 domains. The level of significance was set at 5% in all analyses.

## Results

-Case series

**Figure 2:**
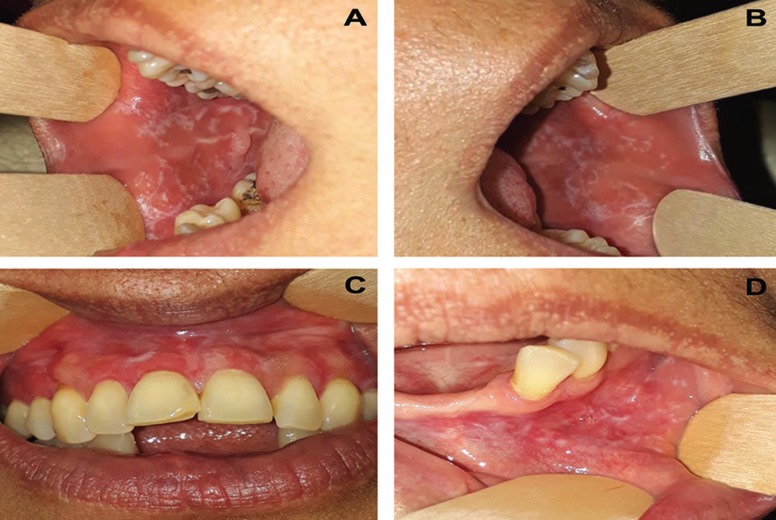
Clinical aspects of reticular oral lichen planus at multiple symmetric bilateral sites. (A, B) Interlaced white lines forming striae (Wickham striae) located in the right and left jugal mucosa. (C, D) Whitish lines located in the upper and lower gingiva.

**Figure 3:**
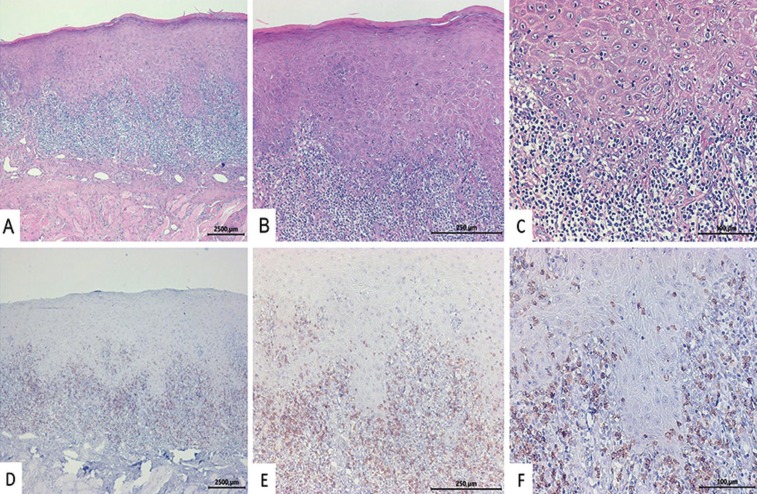
Oral lichen planus exhibiting lymphocytic infiltrate arranged in a band and located in the lamina propria, with destruction of basal keratinocytes, lymphocyte exocytosis, and absence of epithelial dysplasia (A-C). Photomicroscopes images illustrate predominantly CD8+ lymphocytes in the inflammatory infiltrate (D-F). Hematoxylin and eosin: A = 5×, B = 10× and C = 20×; Immunohistochemistry: D = 5×, E = 10× and F = 20×.

Forty-one individuals were considered to have a definitive diagnosis of OLP according to the clinical and histopathological criteria established by the AAOMP/2016 (Figs. 1-3), representing about 0.33% of the individuals whose biopsy specimens (n=12088) were analyzed by the laboratory of Oral and Maxillofacial Pathology between 1998 and 2018.

Women were more affected (n=29; 70.7%) than men (n=12; 29.3%). Patient age ranged from 22 to 72 years, with mean age being 45±13.6 years for women and 42±13.6 years for men ( Table 1). The reticular (n=23, 56.1%) and erosive (n=14, 34.3%) types were the most frequent. OLP affected different regions, the buccal mucosa being the most frequently affected site (n=28 lesions, 68.2%). Clinical manifestations at a single bilateral and symmetric lesion occurred in 51.9% (n=22) of the individuals, and symmetric lesions at multiple sites of the buccal mucosa occurred in 46.4% (n=19) of the patients ( Table 1, Fig. 2).

Regarding symptomatology, there were 23 cases of symptomatic OLP with pain (56.1%); of these 88.9% exhibited the atrophic/erosive/bullous type. Therapy with a dexamethasone mouthwash (0.1 mg/mL; 12/12 hours) was implemented in all symptomatic cases and topical treatment with triamcinolone acetonide (1 mg/g; 8/8 hours) was prescribed for asymptomatic patients (n=8, 19.5%). Of the 31 medicated patients, 21 did not improve with the medication (67.7%). Data regarding recurrence of the lesions were obtained for 27 patients, with nine of them (33.3%) suffering a relapse within up to one month after the initial visit and the others with two months to more than one year. The mean follow-up was of 10.6±25.1 months (range: 1-96 months) ( Table 1).

-Quality of life (OHIP-14)

Thirteen of the patients with a definitive diagnosis of OLP (31.7%) came to the CGDB for clinical evaluation and filling of the questionnaire; 53.8% (n=7) of them having the reticular clinical type and 46.2% (n=6) the erosive type. Patients with the erosive clinical type of OLP, with multiple symmetric/bilateral and with no response to corticotherapy had higher values in the functional limitation, pain and discomfort domains. Social disability was worse in patients with multiple symmetric/bilateral lesions ( Table 2).

**Table 2:**
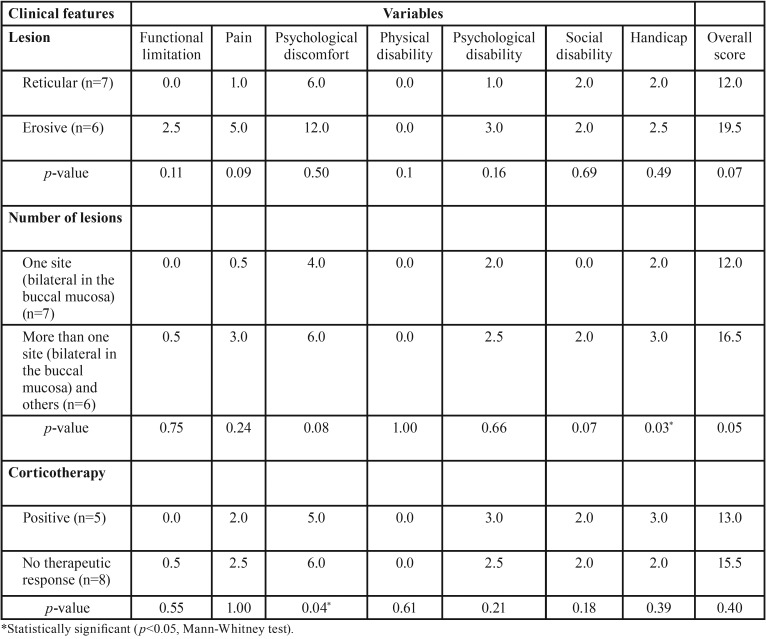
Mean scores of OHIP subscales according to the clinical features of oral lichen planus, anatomical location and corticotherapy.

Comparison of the groups revealed that patients with OLP at multiple symmetric/bilateral sites had worse scores for the total disability domain than patients with lesion at a single bilateral site (*p*=0.03). In addition, patients who did not respond to corticotherapy for OLP symptoms had significantly worse results in the psychological discomfort domain than patients who responded to corticotherapy (*p*=0.04). The results obtained for the remaining domains were similar for all groups (*p*>0.05) ( Table 2).

## Discussion

Clinicians, maxillofacial surgeons and oral and maxillofacial pathologists face a constant challenge in the diagnosis of OLP since the clinical and microscopic characteristics of the condition are similar to those of other diseases such as oral leukoplakia, oral frictional hyperkeratosis, oral pemphigus, and mainly oral lichenoid reaction (5,11,16). Other factors that appear to influence the diagnosis of OLP are the varied clinical forms of the disease, i.e., reticular, erosive, atrophic, bullous, the multiple anatomical regions affected, the still undefined etiopathogenesis, as well as the histopathological characteristics, which are influenced by disease activity at the time when a biopsy is taken (5,11). Cheng *et al.* (5) have reported that probably no other disease in the oral and maxillofacial pathology field has caused such controversy and debate regarding its diagnosis as OLP, underscoring the importance of the association of clinical and microscopy data for the investigators.

The present case series revealed that OLP was more frequent among women in their forties, with presentation in the reticular form and with lesions in the buccal mucosa. Worldwide studies of individuals with OLP have reported similarities in the clinical and demographic profile as demonstrated in  Table 3,  Table 3 continue,  Table 3 continue-1,  Table 3 continue-2. These results may be of aid for clinicians within the context of OLP identification, since the diagnostic hypothesis could be raised according to this clinical and demographic profile. However, according to the criteria established by the AAOMP/2016 (5), the presence of specific clinical and microscopic parameters is necessary for a definitive diagnosis of OLP, and these parameters should be correlated at the time of diagnosis. In contrast to previously published studies (21-23), the present report is the first Brazilian survey to use the parameters established by the AAOMP/2016 for a final diagnosis of this condition.

**Table 3:**
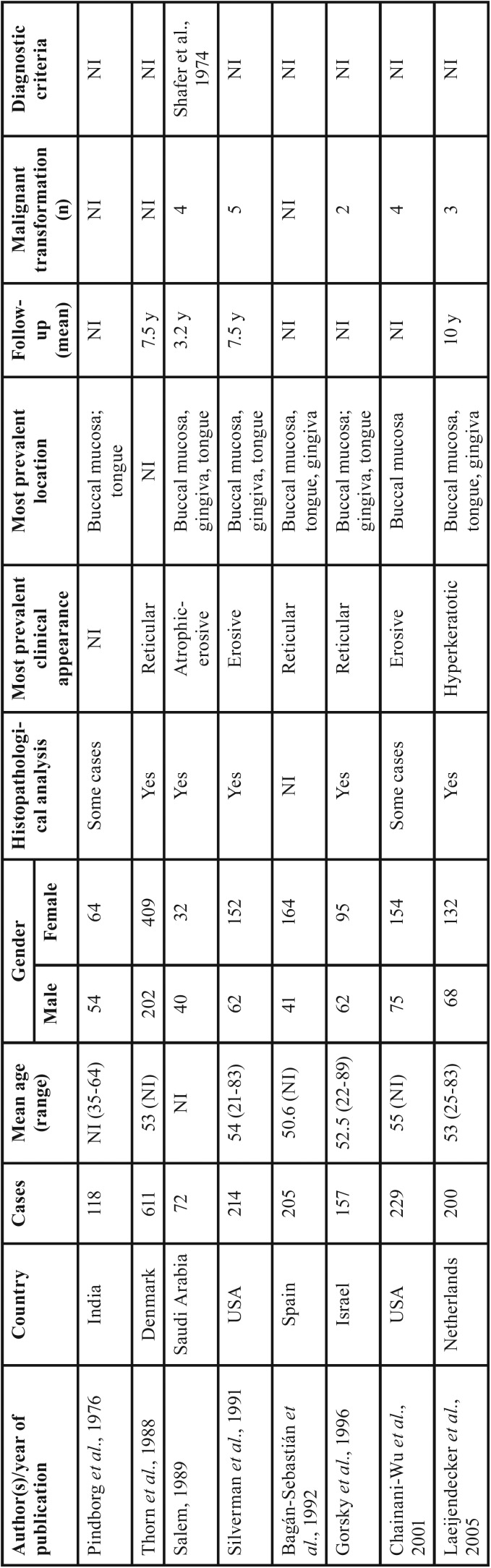
Studies of oral lichen planus lesions in different geographic regions of the world.

**Table 3 continue:**
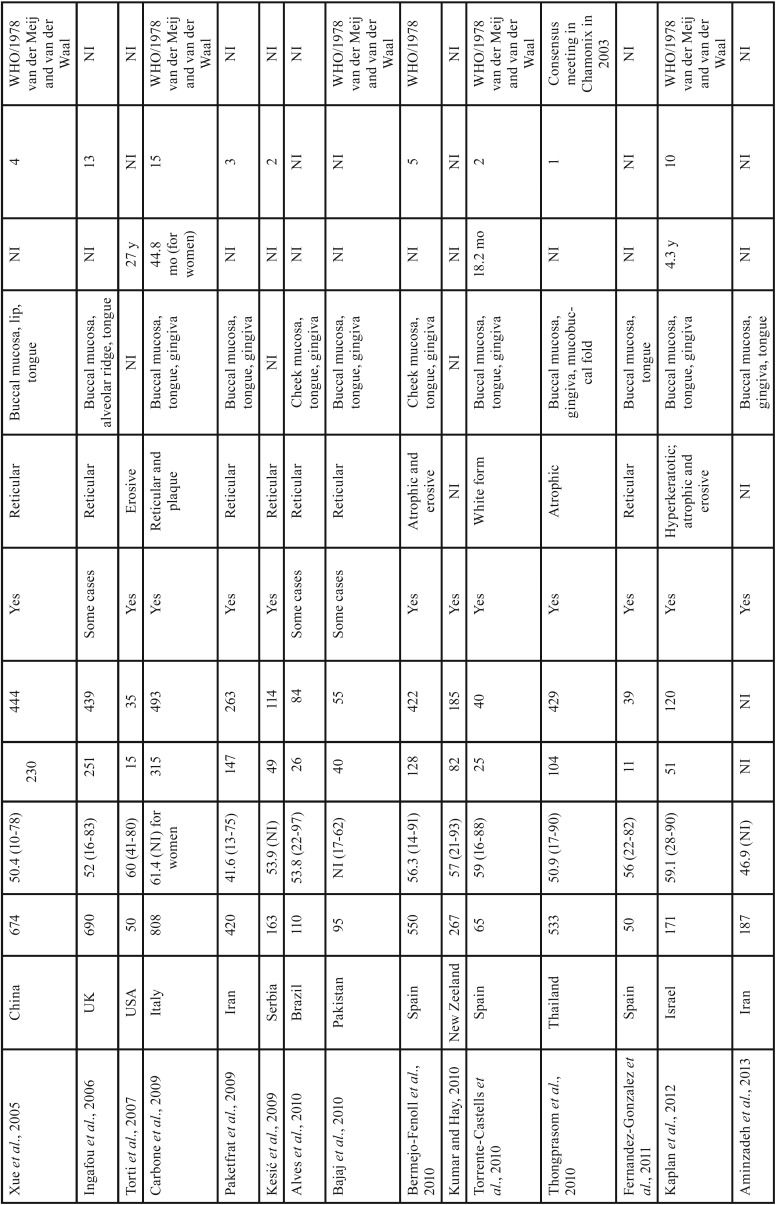
Studies of oral lichen planus lesions in different geographic regions of the world.

**Table 3 continue-1:**
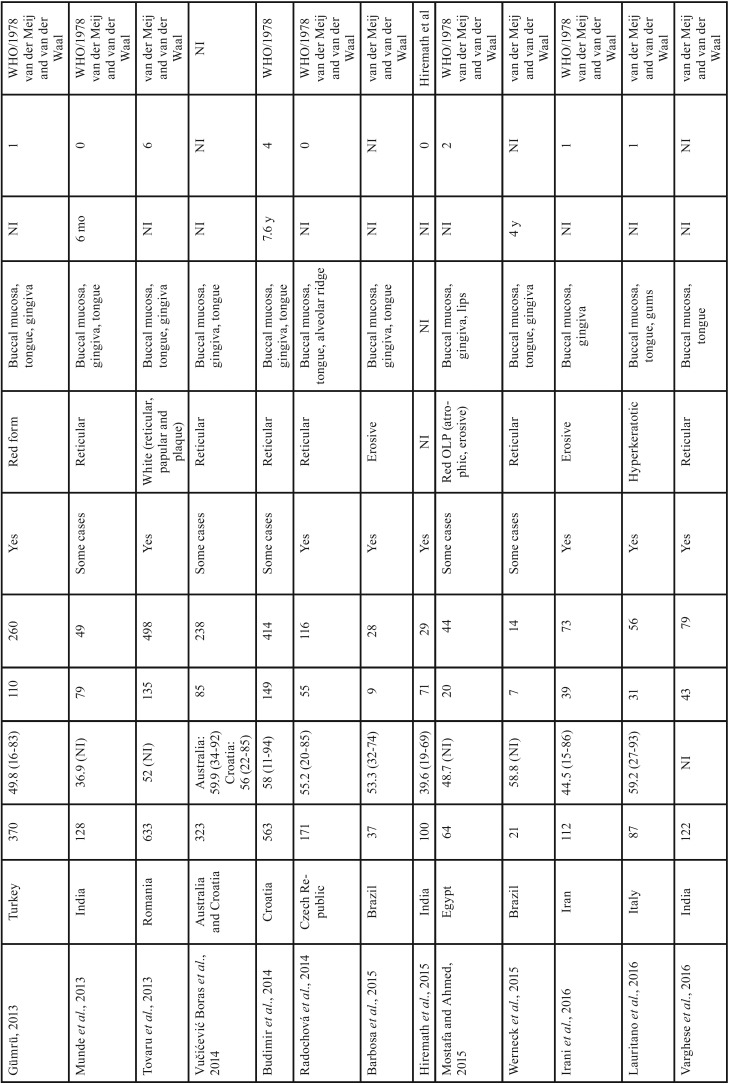
Studies of oral lichen planus lesions in different geographic regions of the world.

**Table 3 continue-2:**
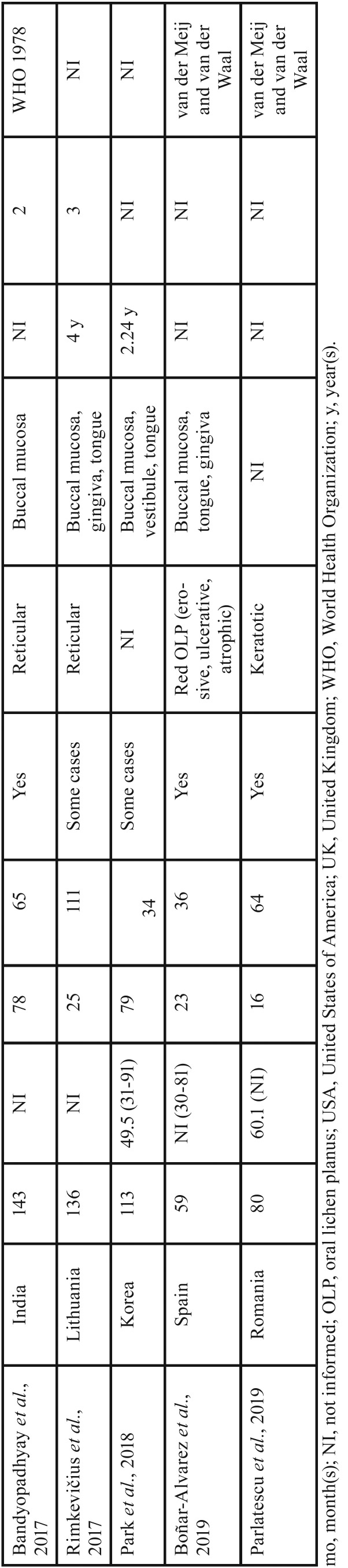
Studies of oral lichen planus lesions in different geographic regions of the world.

Herein, 88.9% of the cases with painful symptoms were of the erosive type. This was also observed in other studies, in which OLP cases showed features ranging from no symptoms to mild burning sensations and to extreme pain degrees observed in the erosive form (5,11).

Dexamethasone and triamcinolone were the treatments used in the present study. Of the 31 patients treated, 67.7% did not respond to the medication, demonstrating the need for a more effective treatment of OLP since the fact that its etiopathogeny has not been fully elucidated only permits treatment limited to the relief of symptoms with the use of local or systemic immunosuppressors (4,5,9,11,26). Although the treatment of asymptomatic individuals is a controversial issue and, to date, does not have guidelines that contribute to clinical decision making, individuals with eritroplasic lesions are medicated preventively in order to offer greater comfort (5,26).

In this study, there was no case of malignant transformation; however, despite doubts about the potential for malignant transformation of OLP, systematic reviews have detected that the rate of such occurrence ranges from 0 to 3.5% and the overall rate of transformation is 1.09% (27-29).

In view of the clinical implications of OLP, quality of life has been increasingly recognized as an important indicator of the need for more effective treatments. As an example, Karbach *et al.* (13) used OHIP-14 and concluded that the life of patients with symptomatic OLP who did not respond to corticotherapy was more affected by the disease. In agreement with the present findings, Alves *et al.* (12) observed that, as a consequence of long and unsuccessful treatment, patients with OLP become emotionally unstable and their quality of life is affected.

In addition, on the basis of our OHIP-14 findings, we observed that patients with lesions at multiple symmetric/bilateral sites and with the erosive clinical type of the disease had worse scores in the functional limitation, physical pain, psychological discomfort, psychological disability, and mainly total disability domains compared to patients with lesions at a single bilateral site and with the reticular clinical type. These results suggest that the number and the clinical type of these lesions have a negative impact on the life of the patients in terms of their concerns, social interaction and performance of daily activities, as demonstrated by Zucoloto *et al.* (11), who reported that the severity of OLP was proportional to its impact on quality of life in a Brazilian case series. However, large case series are needed in order to reinforce our findings.

The present investigation has some limitations that should be recognized. The first regards the sample size and the low response rate in the follow-up. The second is the retrospective nature of the study. Another limitation was the lack of a group of individuals without OLP. The presence of a control group would have become the assessment of the impact of OLP on quality of life more realistic. Finally, the diagnosis of OLP by immunofluorescence (16) was not carried out herein due to its high cost and to the fact that the sample mostly consisted of individuals referred by public health services, where treatment fees are not required.

## Conclusions

In summary, middle-aged female patients with a reticular clinical presentation and lesions in the buccal mucosa were more frequent in this case series, in agreement with the literature. It is important to demonstrate that the standardization and association of clinical and histopathological criteria are important for the diagnosis, and mainly for a safe treatment of patients with this disease. In addition, the more severe clinical forms of the disease that did not respond to drug therapy seem to have a negative impact on the quality of life of the patients, thus further supporting the need for the implementation of other treatments of OLP.
